# *exprso*: an R-package for the rapid implementation of machine learning algorithms

**DOI:** 10.12688/f1000research.9893.2

**Published:** 2017-12-06

**Authors:** Thomas Quinn, Daniel Tylee, Stephen Glatt

**Affiliations:** 1 Bioinformatics Core Research Facility, Deakin University, Victoria, Australia; 2 PsychGENe Lab, SUNY Upstate Medical University, Syracuse, USA

**Keywords:** R, package, machine learning, classification, cross-validation, machine learning, supervised, unsupervised, genomics, prediction

## Abstract

Machine learning plays a major role in many scientific investigations. However, non-expert programmers may struggle to implement the elaborate pipelines necessary to build highly accurate and generalizable models. We introduce *exprso*, a new R package that is an intuitive machine learning suite designed specifically for non-expert programmers. Built initially for the classification of high-dimensional data, *exprso *uses an object-oriented framework to encapsulate a number of common analytical methods into a series of interchangeable modules. This includes modules for feature selection, classification, high-throughput parameter grid-searching, elaborate cross-validation schemes (e.g., Monte Carlo and nested cross-validation), ensemble classification, and prediction. In addition, *exprso *also supports multi-class classification (through the 1-vs-all generalization of binary classifiers) and the prediction of continuous outcomes.

## Introduction

Supervised machine learning has an increasingly important role in biological studies. However, the sheer complexity of machine learning pipelines poses a significant barrier to expert biologists unfamiliar with the intricacies of machine learning. Moreover, many biologists lack the time or technical skills necessary to establish their own pipelines. Here, we discuss the exprso package, a framework for the rapid implementation of high-throughput machine learning, tailored specifically for use with high-dimensional data. As such, this package aims to empower investigators to execute state-of-the-art binary and multi-class classification, as well as regression, with minimal programming experience necessary.

Although R offers a tremendous number of high-quality machine learning packages, there exists only a handful of fully integrated machine learning suites for R. Of these, we recognize here the caret package which offers an expansive toolkit for both classification and regression analyses ^1^. Otherwise, we acknowledge the RWeka package which provides an API to the popular Weka machine learning suite, originally written in Java ^2^. While these packages have a vast repertoire of functionality, we believe the exprso package has some advantages.

First, this package employs an object-oriented design that makes the software intuitive to lay programmers. In place of a few, elaborate functions that offer power at the expense of convenience, this package makes use of more, simpler functions whereby each constituent event has its own method that users can combine in tandem to create their own custom analytical pipeline. Second, this package contains single functions that execute elaborate high-throughput machine learning pipelines. These, coupled with special argument handlers, manage sophisticated pipelines such as high-throughput parameter grid-searching, Monte Carlo cross-validation ^3^, and nested cross-validation ^4^. Moreover, users can embed these high-throughput modules (e.g., parameter grid-searching) within other modules (e.g., Monte Carlo cross-validation), allowing for infinite possibility. In addition, this package provides an automated way to build ensembles from the results of these high-throughput modules.

In addition, this package facilitates multi-class classification by generalizing binary classification methods to a multi-class context. Specifically, this package automatically executes 1-vs-all classification and prediction whenever working with a dataset that contains multiple class labels. Moreover, this package provides a specialized high-throughput module for 1-vs-all classification with individual 1-vs-all feature selection, an alternative to conventional multi-class classification that has been reported to improve results (at least in the setting of 1-vs-1 multi-class support vector machines) ^5^. The exprso package also supports the prediction of continuous outcomes.

While we acknowledge that premier machine learning suites, like caret, may surpass our package in the breadth of their functionality, we do not intend to replace these tools. Rather, we developed exprso as an adjunct, or alternative, tailored specifically to those with limited programming experience, especially biologists working with high-dimensional data. That said, we hope that even some expert programmers find value in our software.

## Methods

### Implementation

This package uses an object-oriented framework for machine learning. In this paradigm, every unique task, such as data splitting (i.e., creating the training and validation sets), feature selection, and model construction, has its own associated function, called a method. These methods typically work as wrappers for other R functions, structured so that the objects returned by one method will feed seamlessly into the next method.

In other words, each method represents one of a number of analytical modules that provides the user with stackable and interchangeable data processing tools. Examples of these methods include wrappers for popular feature selection methods (e.g., analysis of variance (ANOVA), recursive feature elimination ^6, 7^, empiric Bayes statistic ^8^, minimum redundancy maximum relevancy (mRMR) ^9^, and more) as well as numerous models (e.g., support vector machines (SVM) ^10^, neural networks ^11^, deep neural networks ^12^, random forests ^13^, and more).

We have adopted a nomenclature to help organize the methods available in this package. In this scheme, most functions have a few letters in the beginning of their name to designate their general utility. Below, we include a brief description of these function prefixes along with a flow diagram of the available methods.

- **array:** Modules that import data stored as a data.frame, ExpressionSet object, or local text file. Alternatively, the exprso function imports data in *x*, *y* format (recommended for most users).
- **mod:** Modules that modify the imported data prior to building models.
- **split:** Modules that split these data into training and validation (or test) sets.
- **fs:** Modules that perform feature selection.
- **build:** Modules that build models and ensembles.
- **predict:** Modules that deploy models and ensembles.
- **calc:** Modules that calculate model performance, including area under the receiver operating characteristic (ROC) curve (AUC).
- **pl:** Modules that manage pipelines, including high-throughput parameter grid-searches, Monte Carlo cross-validation, and nested cross-validation.
- **pipe:** Modules that filter the pipeline results.

**Figure 1.**
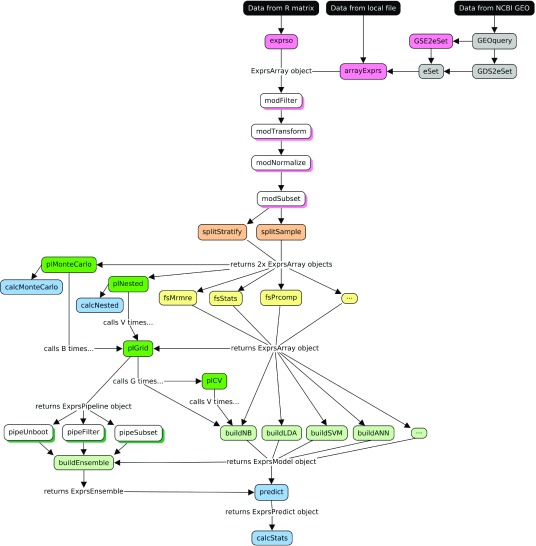
A directed graph of all modules included in the exprso package and how they might relate to each other in practice. Elements colored grey exist outside of this package and instead refer to natively compatible components from the GEOquery ^14^ and Biobase ^15^ packages. Elements colored black indicate possible data sources.

We refer the reader to the package vignette, “An Introduction to the exprso Package,” hosted with the package on the Comprehensive R Archive Network (CRAN), for a detailed description of the functions available from this package ^16^.

### Operation

Specific computer hardware requirements will depend on the size of the dataset under study and the functions used. For the most part, however, a standard laptop computer with the latest version of R installed will handle most applications of the exprso package.

## Use cases

To showcase this package, we make use of the publicly available hallmark Golub 1999 dataset to differentiate (i.e., classify) acute lymphocytic leukemia (ALL) from acute myelogenous leukemia (AML) based on gene expression as measured by microarray technology ^17^. We begin by importing this dataset as an ExpressionSet object from the package GolubEsets (version 1.16.0) ^18^. Then, using the arrayExprs function, we load the ExpressionSet object into exprso. Note that, alternatively, one could use the exprso function to import the data in *x*, *y *format (recommended for most users).

Next, using the modFilter, modTransform, and modNormalize methods, we threshold filter, log2 transform, and standardize the data, respectively, reproducing the pre-processing steps taken by the original investigators ^19^. To keep the code clear and concise, we make use of the %>% function notation from the magrittr package ^20^. In short, this function passes the result from the previous function call to the first argument of the next function, known as piping.



                    **library**(exprso)

                    **library**(golubEsets)

                    **library**(magrittr)

                    **data**(Golub_Merge)

                    **array** <-
  arrayExprs(Golub_Merge,
             colBy = "ALL.AML",
             include = 
                    **list**("ALL","AML"))%>%
  modFilter(20, 16000, 500, 5) %>%
  modTransform %>%
  modNormalize


Then, using the splitSample method, one of the split methods shown in the above diagram, we partition the data into a training and a test set through random sampling without replacement. Next, we perform a series of feature selection methods on the extracted training set. Through the fs modules fsStats and fsPrcomp, we pass the top 50 features as selected by the Student’s t-test through dimension reduction by principal components analysis (PCA).


splitSets <- splitSample(
                    **array**, percent.include = 67)

                    **array**.fs <-
  trainingSet(splitSets) %>%
  fsStats(how = "t.test") %>%
  fsPrcomp(top = 50)



With feature selection complete, we can build the classifier model. For this example, we use the buildSVM method to train a linear kernel support vector machine (SVM) (with default parameters) using the top 5 principal components. Then, we deploy the trained machine on the test set from above. Note that, by design, each feature selection event, including the rules for dimension reduction by PCA, gets passed along automatically at every step up until model deployment. This ensures that the test set always undergoes the same feature selection history as the training set. The calcStats function allows us to calculate classifier performance as sensitivity, specificity, accuracy, or area under the curve (AUC) ^21, 22^.


pred <-
  
                    **array**.fs %>%
  buildSVM(top = 5, kernel = "linear") %>%
  
                    **predict**(testSet(splitSets))
calcStats(pred)


When constructing a model using a build module, we can only specify one set of parameters at a time. However, investigators often want to test models across a vast range of parameters. For this reason, we provide methods like plGrid to automate high-throughput parameter grid-searches. These methods not only wrap model construction, but also model deployment. In addition, they accept a fold argument to toggle leave-one-out or v-fold cross-validation.

Below, we show a simple example of parameter grid-searching, whereby the top 3, 5, and 10 principal components, as established above, get used to construct linear and radial kernel SVMs with costs of 1, 101, and 1001. In addition, we calculate a (biased) 10-fold cross-validation accuracy to help guide our choice of the final model parameters. (Note that we call this accuracy biased because we are performing cross-validation on a dataset that has already undergone feature selection. Although this approach gives a poor assessment of absolute classifier performance ^23^, it may still have value in helping to guide parameter selection in a statistically valid manner. As an alternative to this biased cross-validation accuracy, users can instead call the plNested method in which feature selection is performed anew with each data split that occurs during the leave-one-out or v-fold cross-validation.)


pl <-
  plGrid(
                    **array**.fs, testSet(splitSets),
         top = 
                    **c**(3, 5, 10),
         how = "buildSVM",
         kernel = 
                    **c**("linear", "radial"),
         cost = 
                    **c**(1, 101, 1001),
         fold = 10)


Finally, we show an example for the plMonteCarlo method, an implementation of Monte Carlo cross-validation. Compared to the plGrid method which iteratively builds and deploys models on a validation (or test) set, plMonteCarlo wraps multiple iterations of data splitting, feature selection, and parameter grid-searching. The final result therefore contains a summary of the model performances as measured across any number of bootstraps carved out from the initial dataset. Argument handler functions help organize the arguments supplied to the splitting, feature selection, and high-throughput methods of the plMonteCarlo method call. (Note that when using the Monte Carlo cross-validation method (or any of the other pl modules), the user may iterate over any build method provided by exprso, not only buildSVM. This includes the buildDNN method for deep neural networks as implemented via h2o ^12^. Also note that the user can embed other cross-validation methods, such as another Monte Carlo or nested method, within the cross-validation method call, allowing for endless combinatory possibility.)

In the first section of the code below, we define the argument handler functions for the plMonteCarlo call. As suggested by their names, the ctrlSplitSet, ctrlFeatureSelect, and ctrlGridSearch handlers manage arguments to data splitting, feature selection, and high-throughput grid-searching, respectively. In this example, we set up arguments to split the unaltered training set through random sampling with replacement, perform the two-step feature selection process from above, and run a high-throughput parameter grid-search with biased cross-validation. The unaltered dataset is processed this way 10 times, as directed by argument B.


ss <-
  ctrlSplitSet(func = "splitSample",
               percent.include = 67,
               
                    **replace** = TRUE)
fs <-
  
                    **list**(ctrlFeatureSelect(func = "fsStats",
                         how = "t.test"),
       ctrlFeatureSelect(func = "fsPrcomp",
                         top = 50))
gs <-
  ctrlGridSearch(func = "plGrid",
                 top = 
                    **c**(3, 5, 10),
                 how = "buildSVM",
                 kernel = 
                    **c**("linear", "radial"),
                 cost = 
                    **c**(1, 101, 1001),
                 fold = 10)
boot <-
  plMonteCarlo(trainingSet(splitSets),
               B = 10,
               ctrlSS = ss,
               ctrlFS = fs,
               ctrlGS = gs)


Optionally, one can use these results to build an ensemble of the best models from each bootstrap, then deploy that censemble on the withheld test set. Analogous to how random forests will deploy an ensemble of decision trees ^24^, this method, which we dub "random plains", will deploy an ensemble of SVMs.


ens <- buildEnsemble(boot, colBy = "valid.acc", top = 1)
pred <- 
                    **predict**(ens, testSet(splitSets))


The above procedure, including building and deploying ensembles, also works for multi-class classification and continuous outcome prediction. We refer the reader to the package vignettes, “An Introduction to the exprso Package” and "Advanced Topics for the exprso Package", both hosted with the package on the Comprehensive R Archive Network (CRAN), for a detailed description of all methods included in this package ^16^.

## Summary

Here we introduce the R package exprso, a machine learning suite tailored specifically to working with high-dimensional data. Unlike other machine learning suites, we have prioritized simplicity of use over expansiveness. As such, exprso provides a fully interchangeable and modular programming interface that allows for the rapid implementation of classification and regression pipelines. We have included in this framework functions for executing some of most popular feature selection methods and machine learning algorithms. In addition, exprso also contains a number of modules that perform high-throughput parameter grid-searching in conjunction with sophisticated cross-validation schemes. Owing to its ease-of-use and extensive documentation, we hope exprso will serve as a helpful resource, especially to scientific investigators with limited prior programming experience.

## Software availability

Software available from: http://cran.r-project.org/web/packages/exprso/

Latest source code: http://github.com/tpq/exprso

Archived source code as at time of publication: http://dx.doi.org/10.5281/zenodo.1069113 ^25^

Software license: GNU General Public License, version 2 
