# Supplementary material for: exprso: an R-package for the rapid implementation of machine learning algorithms
Source: F1000Res. 2017 Dec 6;5:2588. Originally published 2016 Oct 27. [Version 2] doi: 10.12688/f1000research.9893.2 (PMC5832912; doi:10.12688/f1000research.9893.2)
Supplement: Peer review history

# Peer review: Referee response for version 2

**DOI:** 10.5256/f1000research.14476.r28684

**Published:** 2018-02-28

**Authors:** Henry Löffler-Wirth

**Affiliations:** 1 Leipzig University, Interdisciplinary Centre for Bioinformatics, Leipzig, 04107, Germany

The authors addressed my comments in their new manuscript and software versions.

 In particular, the program seems more convenient in its current state, and it is also under continuous development.

I have read this submission. I believe that I have an appropriate level of expertise to confirm that it is of an acceptable scientific standard.

# Peer review: Referee response for version 1

**DOI:** 10.5256/f1000research.10664.r20319

**Published:** 2017-02-17

**Authors:** Henry Löffler-Wirth

**Affiliations:** 1 Leipzig University, Interdisciplinary Centre for Bioinformatics, Leipzig, 04107, Germany

The authors present a new tool integrating established algorithms into one R package. This tool is intended to provide access to state-of-the-art statistical analysis to users with limited programming experience. As the manuscript is rather a package vignette than an independent article, I focus my review mainly on the software and usability.

 In general, the intention of providing comprehensive analysis options for non-experts is desirable, however a bit overachieving. In particular, I have several years of programming experience in R, but I was not able to apply the presented methods to another (multiclass) data with acceptable effort of time. Non-expert programmers, as biologists or doctors, also will not be able to correctly use the software in its present form.

 I therefore recommend extensive improvement of usability, user guidance and error feedback. I also acknowledge how challenging implementation of such tools is and encourage the authors to continue development of “exprso”.

 Particular comments:

  

- The *arrayExps*-function should accept also standard matrix containing expression values, the groups may then be given as character vector.
- Handling of most functions can be improved by providing standard parameter values, e.g:

1. in *arrayExps* (may simply include all groups)
2. *percent.include *in *splitSampe* (66%)
3. and so on

- Help the users to find errors in their function calls by giving appropriate warning/error messages. E.g. “fsStats cannot be applied in multi-class studies”. No non-programmer will know what an “inherited method” is.
- Addressing the same point: *fsANOVA* returned with an error not clear to me (my data includes 4 groups):

"contrasts can be applied only to factors with 2 or more levels"

- I found no information about this in the help file of the fs-methods. In general, documentation should be split into one individual file for each function.
- In the manuscript and the package vignette, the authors provide an overview figure of all functions implemented in the package. I suggest revision of this figure: a more systematic flowchart layout of the functions graph would help to find the starting point (GEO, local file, R matrix) and to follow the analysis workflow. Top-left part of the figure is not clear to me.

I have read this submission. I believe that I have an appropriate level of expertise to confirm that it is of an acceptable scientific standard, however I have significant reservations, as outlined above.

# Response

**Published:** 2017-12-07

**Authors:** Thomas Quinn

**Affiliations:** Deakin University, Australia

Dear Henry,

Thank you so much for taking the time to perform a detailed and critical review of the software. I regret that professional obligations have delayed me from addressing your feedback in a timely manner. However, I am pleased to say that I have heavily revised the exprso package, incorporating most, if not all, of your suggestions.

Key changes include:

* Easier data import with the `exprso` function which imports data in x, y format

* Software now supports continuous outcome prediction (when importing data via `exprso`)

* More default values (e.g., split modules) to simplify user experience

* Created custom errors for every split, feature selection, build function. Most other functions now have custom errors as well

* Documentation is split up by unique function. ?mod, ?split, ?fs, ?build, etc. all open a table of contents that overviews the unique functions available

* Figure 1 simplified with data sources clearly labeled in black

* Manuscript adjusted to reflect other changes

Also, you should not encounter any error with mutli-class classification when importing data using the new `exprso` function.

(PS: I am aware of a trivial warning in this version that is triggered by predict. It is already fixed in the developmental branch on GitHub).

Cheers,

Thom

# Peer review: Referee response for version 1

**DOI:** 10.5256/f1000research.10664.r18380

**Published:** 2016-12-09

**Authors:** Dariusz Plewczynski, Julian Zubek

**Affiliations:** 1 Centre of New Technologies, University of Warsaw, Warsaw, Poland; 2 Center of New Technologies, University of Warsaw, Warsaw, Poland

This is a short software tool article presenting a new R package for implementing machine learning pipelines. According to the authors it is targeted specifically at non-expert programmers analyzing high-dimensional biological data. The article briefly describes design goals and implementation details of the package, and then provides an example of building full machine learning pipeline for well-known microarray data set.

 The main contribution of this work lies in the prepared software and not in the accompanying manuscript. Because of this in the article there are no clear hypotheses nor conclusions. The software package does not provide any novel algorithms or functionalities. It is conceived as a wrapper which should make existing methods more accessible. This goal is of practical rather than scientific nature. Ultimately, the usefulness of the package can only be confirmed by its wider adoption by the users. This situation makes it hard to write a conclusive review.

 I agree with the motivation behind this software. R package infrastructure can be indeed confusing and it is notoriously hard to navigate through it for a beginner programmer. I find the interface adopted by exprso package relatively clean and unambiguous. The way the evaluation methods are implemented is consistent with best machine learning practices. Eventual success of this package depends on how well it will be integrated with other popular packages. I hope that the authors will have the resources for further development of exprso.

 Below I present more detailed comments for the authors:

  

- caret is mentioned as the R package with similar goals to exprso. I would find a more detailed comparison between these two package useful, both in terms of general design and specific use cases.
- It is commendable when newly created packages integrate with existing infrastructure. As I understand, exprso has some limited integration with GEOquery and Biobase packages allowing easy data import. However, after data is loaded all operations use special ExprsArray objects, distinct from native R types such as DataFrame or Matrix. I understand this design choice, but I have to note that it limits interoperability. Should the additional processing in the middle of exprso pipeline be required, data needs to be converted between formats.
- I find the design in which transformations applied on the training set are automatically applied on the testing set controversial. It obscures the pipeline and may not be intuitive for beginner programmers. Moreover, sometimes transformations of the testing set differ slightly from the transformations of the training set.
- It is not obvious to me why simple train-test split is implemented as "split" module and cross-validation as "pl" module. There are not very much different. The way cross-validation is currently implemented does not allow detailed control over individual folds, which is sometimes useful.

We have read this submission. We believe that we have an appropriate level of expertise to confirm that it is of an acceptable scientific standard.
